# A Modular, Reconfigurable Microfabricated Assembly Platform for Microfluidic Transport and Multitype Cell Culture and Drug Testing

**DOI:** 10.3390/mi11010002

**Published:** 2019-12-18

**Authors:** Xin Xie, Sushila Maharjan, Sanwei Liu, Yu Shrike Zhang, Carol Livermore

**Affiliations:** 1 Department of Mechanical and Industrial Engineering, Northeastern University, Boston, MA 02115, USA; xxsteven.ee@gmail.com; 2 Division of Engineering in Medicine, Brigham and Women’s Hospital, Department of Medicine, Harvard Medical School, Cambridge, MA 02139, USA; ruma_sushila@hotmail.com; 3 Research Institute for Bioscience and Biotechnology, Nakkhu-4, Lalitpur 44600, Nepal; 4 MEMS Sensors and Actuators Laboratory, Institute for Systems Research, University of Maryland, College Park, MD 20742, USA; swliu@umd.edu

**Keywords:** modular microassembly, modular microfluidics, microphysiological system, cell culture, drug toxicity assay

## Abstract

Modular microfluidics offer the opportunity to combine the precise fluid control, rapid sample processing, low sample and reagent volumes, and relatively lower cost of conventional microfluidics with the flexible reconfigurability needed to accommodate the requirements of target applications such as drug toxicity studies. However, combining the capabilities of fully adaptable modular microelectromechanical systems (MEMS) assembly with the simplicity of conventional microfluidic fabrication remains a challenge. A hybrid polydimethylsiloxane (PDMS)-molding/photolithographic process is demonstrated to rapidly fabricate LEGO^®^-like modular blocks. The blocks are created with different sizes that interlock via tongue-and-groove joints in the plane and stack via interference fits out of the plane. These miniature strong but reversible connections have a measured resistance to in-plane and out-of-plane forces of up to >6000× and >1000× the weight of the block itself, respectively. The LEGO^®^-like interference fits enable O-ring-free microfluidic connections that withstand internal fluid pressures of >120 kPa. A single layer of blocks is assembled into LEGO^®^-like cell culture plates, where the in vitro biocompatibility and drug toxicity to lung epithelial adenocarcinoma cells and hepatocellular carcinoma cells cultured in the modular microwells are measured. A double-layer block structure is then assembled so that a microchannel formed at the interface between layers connects two microwells. Breast tumor cells and hepatocytes cultured in the coupled wells demonstrate interwell migration as well as the simultaneous effects of a single drug on the two cell types.

## 1. Introduction

The applications of microelectromechanical systems (MEMS) and microfluidic chip technologies have increased significantly with advances in soft lithography [1], system integration [2], and MEMS microassembly [3]. The resulting integration of multiple functions to manipulate, control, and/or analyze tiny volumes of fluids [4,5] have enabled the development of numerous miniature cell-based assay platforms. These platforms, which can control multiple reagents in an automated manner in a single experiment, have been used for studies including genetic analysis [6], high-throughput screening assays for drug discovery [2,7], single-cell analysis [8], bioreactors [9], and biomolecular analysis [10]. Recently, modular microfluidic devices assembled from a variety of individual functional modules have been demonstrated in which each module consists of a unified standard interface for easy assembly [11,12]. Unlike monolithically fabricated microfluidic devices, modular microfluidic devices are assembled from a set of prefabricated units. Each module with a specific function can be reconfigured and modified separately before being integrated to create different multiplexed applications and added functionality [13]. Their ability to be reconfigured, precise control of fluids, rapid sample-processing time, lower sample/reagent consumption, lower cost, and portability have made modular microfluidic devices attractive tools for high-throughput screening assays for drug discovery [5,14] and other applications.

Polydimethylsiloxane (PDMS) is one of the most common elastomeric materials used to fabricate microfluidic devices [15], due to its excellent biocompatibility, transparency, gas permeability, elasticity, modifiable surface chemistry, and ability to reversibly or irreversibly bond to other PDMS surfaces or to other materials including glass and plastics [16,17]. The master molds used to fabricate PDMS devices are commonly made from SU-8, which is a chemically inert and thermally stable acid-catalyzed negative photoresist [18]. SU-8 master molds allow for batch manufacture of high-fidelity PDMS replicas [19].

Several approaches have been employed to fabricate modular microfluidics, including microfabrication techniques [20,21], three-dimensional (3D) printing [12,13], and micromilling of injection molded blocks [11]. In modular microfluidics, PDMS or other polymer materials are typically fabricated into separate modules that are later assembled into a multifunctional system as in [22,23] via a set of predefined connections. 

For connections, interlocking wafer-scale alignment features and wafer-scale packaging features have previously been demonstrated [24,25]. The elastic averaging of integrated alignment features aligns wafers, but it does not fix them in place [26]. LEGO^®^-inspired rivet packaging creates connections at the wafer scale, but the rivet connections do not extend to device-scale assembly [20]. Surface interactions can also enable modular assembly. For example, in the block-by-block micromasonry, dry adhesion temporarily holds each layer of silicon blocks in place until annealing makes the bonds permanent; one challenge of this approach is that each layer requires an anneal [27]. 

For microfluidics, a microfluidic assembly block platform has been demonstrated in which pre-fabricated PDMS blocks containing various flow structures are pressed together and held in place by adhesives [28]. Hsieh et al. developed a LEGO^®^-like swappable fluidic module concept in which modules connect together mechanically in the plane; a functional microfluidic device was fabricated and demonstrated for the synthesis of gold nanoparticles and capillary convective PCR to amplify deoxyribonucleic acid from the Hepatitis B virus [21]. In [12], enclosed-channel modular microfluidic elements were 3D-printed and connected into microfluidic systems via separate pin connectors and O-ring seals. Recently, 3D-printed LEGO^®^-like modular microfluidic devices with open-topped planar channels and PDMS sealing interfaces were developed and used for studying stent degradation and cell cultures [13]. Store-bought LEGO^®^ bricks were micromilled in [11] to create surface channels that were sealed with adhesive film and O-ring seals that connect adjacent blocks. However, creating compact, microfabricated modular microelements with both in-plane and out-of-plane interlocking connections has nonetheless remained a challenge.

Here we present a modular PDMS-enabled molding approach to rapidly fabricate LEGO^®^-like, modular SU-8 blocks via SU-8 master molding combined with lithography. Stackable building blocks of different sizes (that is, blocks with different areas and different numbers of locking features) were fabricated, and their in-plane and out-of-plane locking features enable precision alignment in every direction (x, y, and z). These millimeter-scale modular blocks could be simply and reversibly assembled to create structures of many shapes and sizes, including microfluidic connections and array structures for different cell-based applications such as drug toxicity studies. As the blocks’ connections are made via compact, submillimeter structures, it was possible to make modular microfluidic elements in which the microfluidic features were at a similar length scale to the interconnection features, minimizing the extra volume required to connect the modular blocks. The mechanical strength of the miniature LEGO^®^-like block-to-block interfaces enabled O-ring-free connections between microfluidic channels with leak-free performance to >120 kPa. To test the biocompatibility of this platform, sets of these blocks were first assembled in a single layer to form LEGO^®^-like cell culture plates. Lung epithelial adenocarcinoma cells and hepatocellular carcinoma cells were cultured in the resulting modular microwells with viabilities analyzed, and the in vitro toxicity of acetaminophen to the hepatocellular carcinoma cells was further measured. Double-layered block assemblies were then created in which microwells in the upper block layer were coupled to each other via a microchannel that formed along the interface between the upper and lower block layers. Breast cancer cells and hepatocytes were cultured in separate microwells, and the interwell migration of the cells through the connecting microchannel was characterized. The simultaneous effect of acetaminophen on the two cells types was also evaluated.

## 2. Materials and Methods

### 2.1. Materials and Reagents

SU-8 2150 epoxy photoresist, SU-8 developer, and Omnicoat were obtained from MicroChem, SUEX dry film epoxy photoresist from DJ Microlaminates, PDMS Sylgard silicone elastomer from Dow Corning, and Dulbecco’s modified Eagle medium (DMEM), Dulbecco’s phosphate-buffered saline (DPBS), fetal bovine serum (FBS), trypsin-ethylenediaminetetraacetic acid (trypsin-EDTA), penicillin/streptomycin, 4′,6-diamidino-2-phenylindole (DAPI), Live/Dead^®^ Viability/Cytotoxicity Kit, Alexa Fluor™ 594 Phalloidin, CellTracker™ Green CMFDA Dye, and CellTracker™ CM-DiI from ThermoFisher Scientific. The CellTiter 96^®^ AQueous One Solution Cell Proliferation Assay solution was purchased from Promega.

### 2.2. Fabrication of Devices

**Figure 1:**
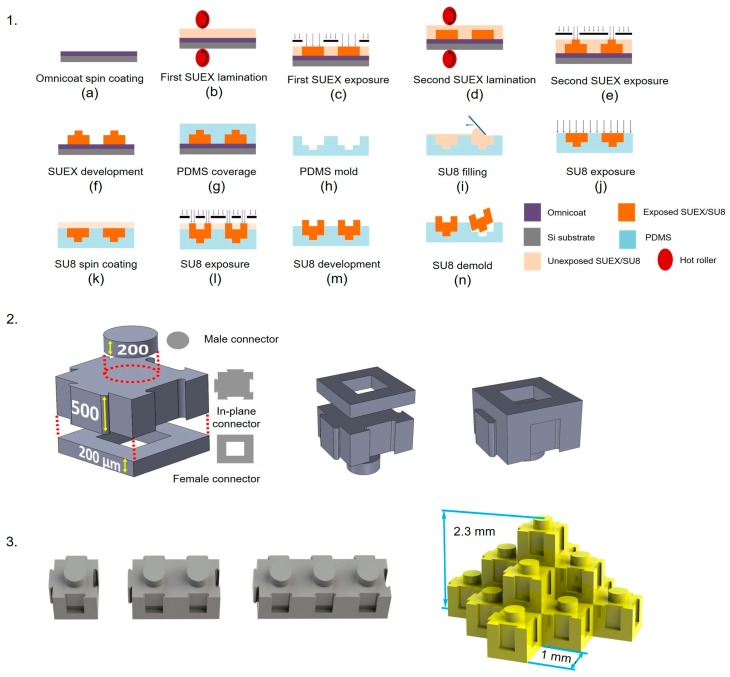
Fabrication and assembly of the micro building blocks. (**1**). Sequence of steps in the fabrication of micro building block elements. (**2**). Schematic diagrams of an exploded view of a micro building block. The male and female connectors are nominally 530 µm and 460 µm across, respectively. (**3**). Schematic diagrams of micro building blocks with different lengths and an assembled pyramid.

A hybrid process that combines molding and photolithography was used to create modular SU-8 elements with recesses on both their upper and lower surfaces (Figure 1); these structures would not be compatible with conventional molding (soft lithography) or with the deposition and patterning of successive layers via conventional photolithography. In this process, a PDMS mold was first cast from a SUEX master. The male connectors were defined by casting SU-8 into the reusable PDMS mold, removing the excess, and exposing the structure. The female connectors are defined on top of the male connector structure using photolithography. The use of molding for the male connectors prevents inadvertent exposure of underlying SU-8 during the subsequent process steps. 

#### 2.2.1. SUEX Master Fabrication

A layer of 15-nm-thick Omnicoat^TM^ was spin-coated on a silicon wafer at 1000 rpm for 10 s for adhesion enhancement. A layer of 500-µm-thick dry-film SUEX epoxy was laminated on the wafer by a hot rolling laminator (SKY 335R6) at 60 °C. The SUEX was patterned using a photolithography process to define the microelements’ in-plane connectors. Then a second layer of 200-µm-thick SUEX was laminated and patterned on the first SUEX layer to define the male connectors. The use of laminated SUEX film offers dimensional uniformity across layers.

#### 2.2.2. PDMS Mold Fabrication

After the SUEX master for the in-plane connectors and the male connectors was made, PDMS prepolymer (monomer:crosslinker = 10:1) was poured on the master and degassed for 10 min, followed by a hotplate bake at 90 °C for 30 min. The cured PDMS was then peeled off of the SUEX master to form the mold.

#### 2.2.3. Microelement Fabrication

The in-plane connectors and the male connectors were made by pouring 20 mL of SU-8 2150 on the PDMS mold. Excess SU-8 was removed by sweeping a razor blade over the PDMS mold. The SU-8 was cured after photolithography exposure. Another layer of approximately 200 µm of SU-8 2150 was spin-coated for 1 min at 2500 rpm. The hot plate bake processes for this layer were extended relative to the manufacturer’s recommended times to account for the combined thermal resistance of the hot plate’s aluminum foil-covered surface and the thick underlying layers of SU-8. The spin-coated layer was soft-baked first at 65 °C for 15 min and then at 95 °C for 2 h, exposed to pattern the female connector and culture well of the block, post-baked for an additional 15 min at 65 °C and an additional 1 h at 95 °C, and then developed. The fabricated SU-8 elements were then demolded from the PDMS and were ready for assembly.

### 2.3. Modular Assembly

The LEGO^®^-like structures interlock in the plane via tongue and groove connections and are connected out of the plane (vertically) by interference fits. Assembly of the present structures was carried out manually but is consistent with pick-and-place techniques. During assembly, the elements (0.9-mm tall, 1-mm wide, and 1-, 2-, or 3-mm long) were positioned so that their in-plane features interlocked, and their vertical features aligned. Pressure was then applied to create interference fits in the vertical direction; the SU-8 was stiff enough to provide adequate compressive forces and deformable enough to accommodate the interference. 

### 2.4. Out-of-Plane Microfluidic Connections

**Figure 2:**
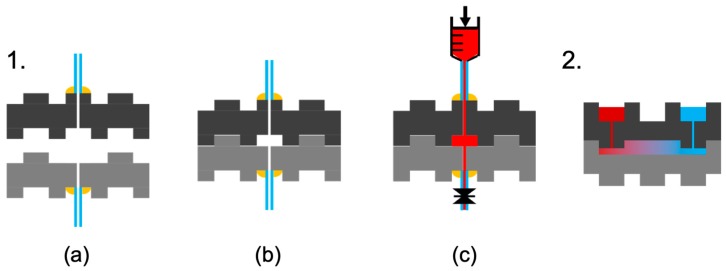
Diagrams of microfluidic testing and of coupled microwell testing. (**1**). For microfluidic testing, tubing is attached on either side of two micro building blocks (**a**); the two micro blocks are pressed together via their interference fits to form an O-ring-free sealed microfluidic system (**b**); and the microfluidic system is tested under pressure from the inlet with a closed outlet (**c**). (**2**). For testing of coupled microwells, a micro block with through holes is pressed to a micro block with a grooved surface to create an enclosed microfluidic channel that couples two microwells from beneath; the color gradient is a schematic representation of mass transport.

To assemble modular microfluidic connections, hollowed blocks were coupled together in the out-of-plane direction via the blocks’ interference fits without the use of O-rings or gaskets (Figure 2-1). Inlet and outlet connections were made to the blocks by adhering silicone rubber tubes with 0.025 in outer diameter and 0.0125 in inner diameter (McMaster Carr) to the blocks’ external surfaces using a thin layer of cyanoacrylate epoxy at the interface with an additional ring of Gorilla Epoxy to surround and seal the connection. Internal pressure was controlled by first flowing water through the device, then sealing the outlet and applying progressively larger calibrated forces to the plunger of a syringe at the inlet. The ability of the connections to withstand pressure was characterized as the maximum applied pressure before the onset of detectable leakage.

### 2.5. Modular Assembly of Coupled Microwells from Double-Layer Blocks

To assemble coupled microwells, a 3 × 1-mm^2^ block that was hollowed at either end to create through connections between the microwells and the opposite surface of the block was connected via the out-of-plane connectors above a second 3 × 1-mm^2^ block (Figure 2-2). In the second 3 × 1-mm^2^ block, the three female connectors were joined together to form a single open-topped flow channel approximately 500-µm wide and 200-µm deep that connected the two ends of the block. This continuous flow channel was created by micromilling out the walls that initially separated the as-fabricated female connectors from each other. When the first block was then placed on top of the second block, the channel in the second block was capped by the bottom surface of the first block to form a sealed connecting channel between the two microwells.

### 2.6. Cell Culture

#### 2.6.1. Cell Culture for Studies of Drug Toxicity in Microwell Arrays

A human adenocarcinomic alveolar basal epithelial cell line, A549 cells, and a human hepatocellular carcinoma cell line, HepG2 cells, were obtained from American Type Culture Collection (ATCC, Manassas, VA, USA). The cells were cultured in DMEM supplemented with 10% FBS and 1% penicillin/streptomycin and incubated in a humidified atmosphere at 5% CO_2_ and 37 °C in an incubator. The medium was replaced every 2–3 days. Cells were split when they reached approximately 80–90% confluency with 0.125% trypsin/EDTA.

Before cell seeding, the single-layer array microchips were sterilized with 70% ethanol and then coated with poly-L-lysine in the wells overnight at 4 °C. Approximately 5 µL of 1 × 10^6^ cells/mL were seeded in each well of the microchip by adding the cell suspension into the well and allowing cells to sediment at the bottom. The microchips with cells were maintained under standard cell culture conditions as described above.

#### 2.6.2. Cell Culture for Studies of Cell Migration and Drug Toxicity in Coupled Microwells

Human hepatocyte-like cells (HepG2-C3A; a clonal derivative of HepG2 that was selected for strong contact inhibition of growth and high albumin production more characteristic of normal hepatocytes than HepG2 cells) and breast cancer cells (4T1) obtained from ATCC were first stained with CellTracker™ Green CMFDA Dye and CellTracker™ CM-DiI, respectively, according to the manufacturer’s instructions. The trypsinized cells were incubated with their respective cell staining dyes for 20 min at 37 °C and were then washed three times with DPBS. Before cell seeding, three replicates of the double-layer block assembly were sterilized with 70% ethanol and then coated with poly-L-lysine in the wells overnight at 4 °C. Approximately 1000 HepG2-C3A cells (or 2 µL of 5 × 10^5^ cells/mL) were then seeded in one microwell at one end of the double-layered, coupled-microwell structure, and approximately 1000 4T1 cells (or 2 µL of 5 × 10^5^ cells/mL) were seeded in the microwell at the opposite end. The system was then incubated at 5% CO_2_ and 37 °C for up to 6 days, and the media were changed every 3 days.

### 2.7. Cell Viability Assays

The cells were cultured in the microchip wells for 96 h, and their viability and proliferation were evaluated by Live/Dead assay using the Live/Dead^®^ Viability/Cytotoxicity Kit according to the manufacturers’ instructions. For the cell viability assay, cells were washed three times with DPBS and incubated with 100 µL/well of the combined Live/Dead assay reagents (2 μM of calcein AM and 4 μM of ethidium homodimer I (EthD-1)) for 20 min at 37 °C in the dark. The cells were then washed with DPBS and observed under fluorescence microscope.

### 2.8. Filamentous Actin (F-actin) Staining

To observe the morphology of HepG2 cells in the single-layer array microchips, the cells were cultured for 120 h under standard cell culture conditions. The cells were then washed with DPBS, fixed with 4% (v/v) paraformaldehyde for 15 min, and permeabilized with 0.1% (v/v) Triton X-100 in PBS for 30 min. The cells were blocked with 5% (w/v) goat serum in DPBS for 1 h, followed by F-actin staining by incubating the cells with Alexa Fluor^®^ 594-phalloidin (1:200 dilution in blocking buffer) for 1 h at room temperature. After washing with DPBS, the nuclei were counter-stained with DAPI (1:1000) for 5 min at room temperature. Finally, the cells were observed under a fluorescence microscope.

### 2.9. Drug Toxicity Assay

#### 2.9.1. Drug Toxicity Assay for Single-Layer Array Microchips

Stock solution (0.5 M) of acetaminophen (APAP) was prepared by dissolving the powder (Sigma-Aldrich) in DPBS:ethanol (1:1 v/v). HepG2 cells were cultured in the microwell array for 48 h as described above and treated with APAP at final concentrations of 0, 5, or 10 mM by replacing the medium in the well. After 12 h, APAP-induced toxicity was assessed by 3-(4,5-dimethylthiazol-2-yl)-5-(3-carboxymethoxyphenyl)-2-(4-sulfophenyl)-2H-tetrazolium (MTS) assay using CellTiter 96^®^ AQueous One Solution Cell Proliferation Assay Kit, according to the manufacturer’s instructions. The media were removed, and the cells were incubated with 80 μL of culture medium and 20 μL of MTS assay solution for 2 h at 37 °C in the dark. The absorbance was measured at 492 nm with a Microplate Reader (Tecan), and the results were expressed as percentage of the control group, which was assumed to be 100%. All the experiments were done in triplicate and were repeated three times.

#### 2.9.2. Drug Toxicity Assay for Double-Layer Connected Microwells

Stock solution (0.5 M) of APAP was prepared as described above. To assess the simultaneous toxicity of APAP to two different cells in coupled microwells, HepG2-C3A cells and 4T1 cells were cultured in the opposite ends of the double-layer coupled microwells for 48 h. The medium in the microwell containing HepG2-C3A cells was replaced to treat the cells with acetaminophen (0, 5, or 10 mM). After 4 h of drug treatment, 100 μL of medium was added, and the cells were incubated for another 24 h. The cells were then analyzed for cell viability using calcein AM, ethidium homodimer I, and MTS assay as described above. In this case the cells were not prelabeled with trackers.

### 2.10. Statistical Analysis

Statistically significant differences between the groups were evaluated by using one-way ANOVA analysis. Data are represented as means (± SEM) and differences were considered significant at *p* < 0.05.

## 3. Results and Discussions

### 3.1. Fabricated Device Structures

**Figure 3:**
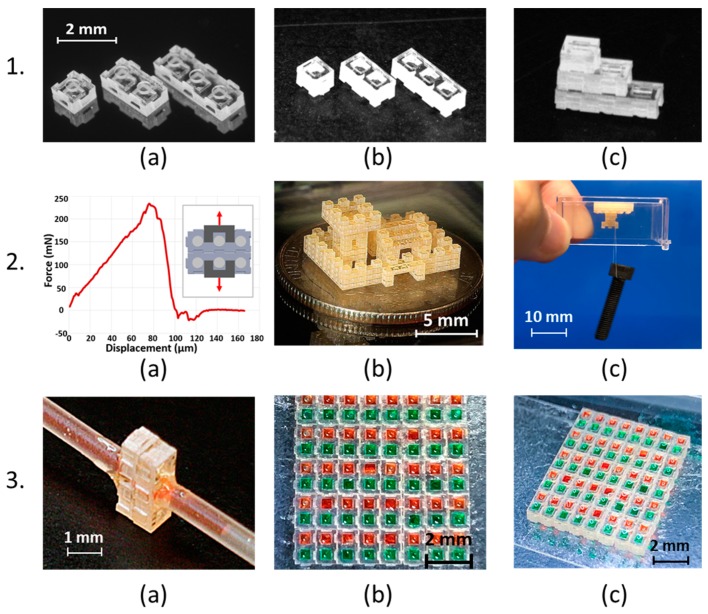
Micrographs and photographs of micro building blocks. (**1**). Optical micrographs of the three elements with footprint dimensions (from left to right) of 1 × 1 mm^2^, 2 × 1 mm^2^, and 3 × 1 mm^2^; all three micrographs have the same scale bar. (**2**). Plot of measured force vs. displacement for two 3 × 1 elements connected via an interlocking in-plane connection (**a**). Photographs of a demonstration structure created by micro building block elements on a US quarter (**b**) and a 4.2 g bolt hanging from the inverted demonstration structure. The weight of the bolt is supported solely by the interference fits (nine of them at the narrowest point) (**c**). (**3**). Photographs of the O-ring-free microfluidic interface testing (**a**), and photographs of the top (**b**) and angled (**c**) view of the assembled microwell chips filled with red- and green-colored liquids.

The micrographs of the as-fabricated elements are shown in Figure 3-1. The final SU-8 structures are transparent but develop a yellow tint upon exposure. The final thickness of the spin-coated female connector layer plus the 700-µm total thickness of the in-plane connector and male connector layers was measured for ten 1 × 2-mm^2^ blocks to confirm the out-of-plane block dimensions. The results range from 880 µm to 910 µm with a mean value of 897 µm, placing an upper bound of 30 µm on the variation in the layer thickness values. The widths of ten nominally 1-mm-wide 1 × 2-mm^2^ blocks were also measured, yielding a range from 0.96 mm to 1.02 mm with an average width of 0.986 mm.

### 3.2. Mechanical Performance of Fabricated Devices

To characterize the separation force of the interlocks, two 3 × 1 elements were connected, mounted in an Instron 5943 benchtop mechanical tester, and loaded in tension. Figure 3-2a plots measured force vs. displacement, with a maximum force capacity of 236 mN corresponding to >6000× the piece’s weight.

A demonstration illustrates the creation of fully 3D architectures with enclosed cavities and reentrant overhangs (Figure 3-2b). In practice, separation force will be reduced when loads are applied unevenly, much as progressive peeling is easier than direct liftoff. These asymmetric loads were mimicked by inverting the demonstration architecture and hanging a series of increasing masses from one side of its overhang (Figure 3-2c). The overhang (supported by nine interference fits) tolerated up to 4.2 g (41 mN) of eccentrically applied load. This load corresponds to >1000× the piece’s weight, as compared with a capacity of ≈400× the weight for conventional building bricks. This is consistent with the expected length scaling of load carrying capacity (which scales with contact area, or length squared) per unit weight (which goes as volume, or length cubed). 

### 3.3. Microfluidic Interface Performance

The O-ring-free microfluidic interface is held together only by the separation resistance of the two LEGO^®^-like interference fits, which are located on either side of the microfluidic interface (Figure 2). The resulting microfluidic interface had a minimum seal width of 320 μm. The interface did not demonstrate observable leakage when water flowed between the inlet and an open outlet (Figure 3-3a). After the outlet was closed to seal the system, the inlet was pressurized by applying increasing forces to the plunger of the inlet syringe. Observable leakage occurred only at measured inlet pressures >160 kPa. Accounting for the friction in the syringe, observable leakage occurred for fluid pressures >120 kPa. Cast elastomer press-fits [21], PDMS interfaces [13], PDMS interfaces with additional adhesive [28], and O-ring-enabled sealing [11,12] have all been used to connect modular microfluidic blocks. However, the sealing of the present blocks under pressure represents the first time that microfabricated microfluidic blocks have achieved sealing using only their own surfaces and the forces provided by their structural connections. The success of these seals can be attributed to the strength of the LEGO^®^-like connection and to the smooth microfabricated surfaces of the blocks.

### 3.4. Cell Behaviors

**Figure 4:**
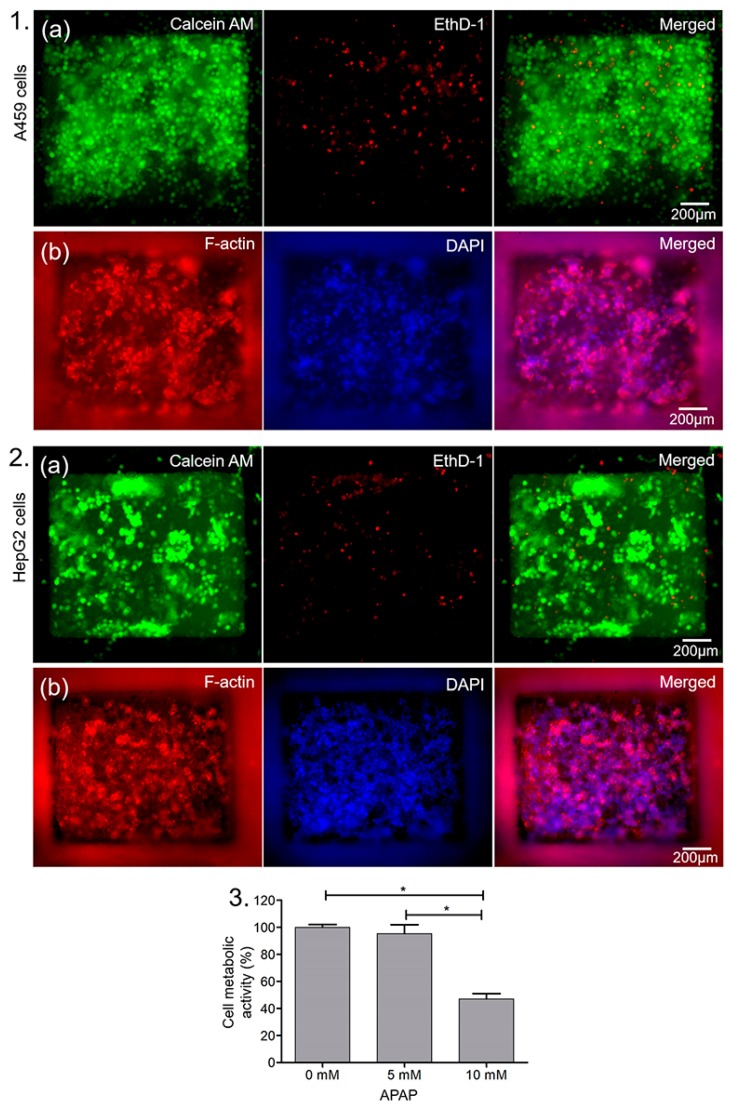
Application of microwell arrays in cell culture and drug testing. (**1**). Viability of A549 cells within modular microwell arrays on day 4 showing (**a**) the live (green) and dead (red) cells, and (**b**) F-actin (red) and nuclei (blue) staining of the cells on day 5. (**2**). Viability of HepG2 cells within modular microwell arrays on day 4 showing (**a**) the live (green) and dead (red) cells, and (**b**) F-actin (red) and nuclei (blue) staining of the cells on day 5. (**3**). Quantitative metabolic activity of HepG2 cells as determined by the 3-(4,5-dimethylthiazol-2-yl)- MTS assay after treatment with indicated doses of APAP after 12 h of exposure. The asterisk represents statistically significant differences (*p* < 0.05).

As miniaturization of conventional cell culture systems through microfluidics and microarray biochips [29,30] attracts growing interest, investigation of short- and long-term viability of cells in microwells becomes a prerequisite for cell function studies and accurate drug screening. Cells were first cultured in the assembled single-layer microwell arrays shown in (Figure 3-3b,c) to demonstrate the biocompatibility of the devices. Viability of the human adenocarcinomic alveolar basal epithelial cell line, A549 cells in the microchips, with initial cell seeding density at approximately 5000 cells/well, was determined separately at 96 h using calcein AM and EthD-1. Calcein AM is a nonfluorescent cell-permeable compound which is hydrolyzed into the green fluorescent anion calcein by intracellular esterase in live cells whereas EthD-1 is a dead cell-specific red fluorescent dye that binds to DNA. Fluorescence images of cells at 96 h showed high cell viability in the microwells, with most of the cells having green fluorescence (Figure 4-1a).

To observe the proliferation and morphology of the cells within the microwells, A549 cells were cultured in the microwells for 120 h and F-actin staining was performed using Alexa Fluor^®^ 594-phalloidin. The cells were counterstained with DAPI for nuclei and imaged using fluorescence microscopy. Actin is one of the most abundant cytoskeletal proteins in all eukaryotic cells, and it can polymerize to form actin filaments. The actin filaments in the cell determine the shape, stiffness, and movement of the cell surface and also facilitate the transduction of mechanical signals as well as generate the intracellular forces required for many cellular functions [31]. Phalloidin possesses a high binding affinity for F-actin subunits and thus fluorescent derivatives of phalloidin remain the gold standard for staining and visualizing cellular F-actin filaments [32]. Fluorescence imaging of F-actin/nuclei-stained cells indicated red staining for F-actin in the cytoplasm while the blue stains for nuclei (Figure 4-1b).

We then evaluated the ability to culture the human liver cancer cell line, HepG2 cells in the microchip wells. Similar to A549 cells, the HepG2 cells also exhibited high viability and good attachment (Figure 4-2). Our data further showed that the hepatocellular carcinoma cells were arranged in cell aggregates as expected, with clusters of cells separated by less-populated areas. Thus, the microchip well arrays were shown to be biocompatible and provide an in vitro environment comparable to that of standardized cell cultures.

**Figure 5:**
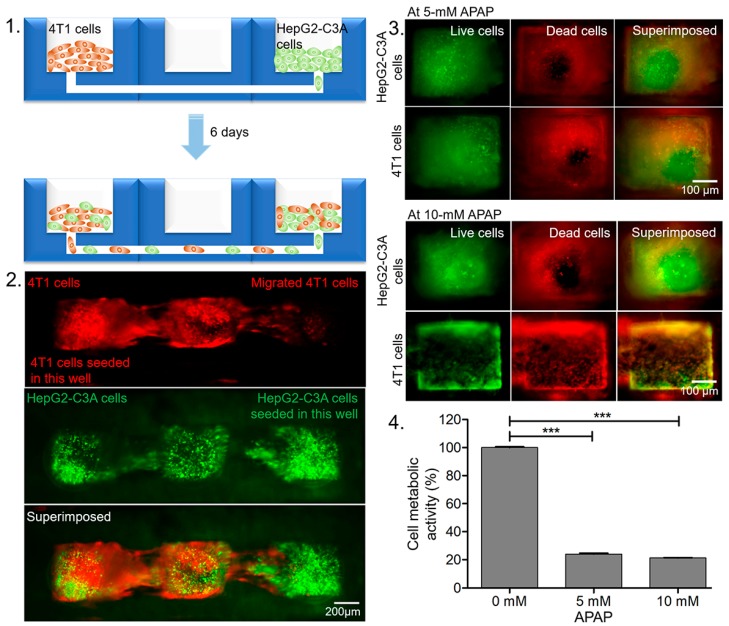
Results of coupled microwell experiments. 1. Schematic showing co-culture of 4T1 cells and HepG2-C3A cells in two microwells that are coupled by an underlying microfluidic channel. 2. Fluorescence micrographs showing cell migration after 6 days of culture. 3. Viability of HepG2-C3A and 4T1 cells in coupled microwells after APAP treatment showing the live (green) and dead (red) cells. 4. Quantitative metabolic activity of the cells measured using the MTS assay after treatment with indicated doses of APAP. The asterisks represent statistically significant differences (*p* < 0.05).

In addition, cell migration is a critical process in cancer progression and the metastatic dissemination of cancer cells. During metastasis, the migration and spread of cancer cells from their initial locations to other sites in the body leads to the development of secondary tumors [33,34]. To study cancer cell migration, we used the double-layered, coupled-microwell assembled device with the human hepatocyte-like cell line, HepG2-C3A cells seeded in one well and the mouse breast cancer cell line, 4T1 cells seeded in the opposite well to visualize the migration of the cells from one well to the other (Figure 5-1). After the 6 days of culture, the modular microfluidic devices were visualized under a fluorescent microscope. The migration of both HepG2-C3A cells and 4T1 cells towards the opposite ends through the underlying channel that coupled the microwells was observed (Figure 5-2), demonstrating the ability of the cells to migrate through the channel for studying cell interactions.

### 3.5. Effects of Acetaminophen on Liver Cell Behaviors

We evaluated the effects of high doses of APAP on the HepG2 hepatocellular carcinoma cells using our single-layer, modular microwell arrays. APAP, also known as paracetamol and N-acetyl-p-aminophenol, is one of the most commonly used antipyretic and analgesic drugs. It is usually safe when taken at low dosage (the United States Food and Drug Administration (FDA)-approved maximum daily dose of APAP is 4 g/day/adult person [33]). However, APAP is the world’s leading cause of severe liver failure due to acute APAP overdose-induced hepatotoxicity [34,35]. Hepatotoxicity and subsequent liver tissue damage following APAP overdoses are due to reactive compounds produced from APAP metabolism in the liver [36]. Most of the studies have focused on high doses (>5 mM) of APAP-induced hepatotoxicity [37,38,39] and nephrotoxicity [40,41,42].

To assess the toxicity of APAP at two different doses in the modular microwell arrays, HepG2 cells were incubated with different concentrations (0, 5, and 10 mM) of APAP, and after 12 h of exposure, cells were treated with the MTS reagent whereby metabolically active cells reduced MTS tetrazolium compound to produce a colored and soluble formazan product in culture medium. The amount of formazan product generated was quantified by measuring the absorbance at 492 nm, which is directly proportional to the number of metabolically active live cells in culture. As shown in Figure 4-3, HepG2 cell viability was decreased by treatment with APAP at each of the two doses. When HepG2 cells were treated with 5-mM APAP, the cell viability was decreased by approximately 10% of the control group whereas cell viability was decreased by more than 50% of the control group when treated with 10-mM APAP.

Similarly, we evaluated the effects of two different high doses of acetaminophen on the cell populations in the double-layered, coupled microwell device after 48 h of culture. The results obtained from both assays indicated that the APAP-treated HepG2-C3A cell chamber exerted toxicity to both HepG2-C3A cells as well as 4T1 cells present in the other chamber (Figure 5-3). The viability of the cells was decreased by 76% when treated with 5 mM of APAP and by 79% when treated with 10-mM APAP, as compared to the control group (Figure 5-4). Thus, our modular microchips customized with migration channel can be used to study the simultaneous effects of a single drug to different cell types.

## 4. Conclusions

In conclusion, we presented a significant advance in modular manufacture of 3D microsystems based on strong but reversible, out-of-plane interference fits between elements inspired by LEGO^®^ building toys. The in-plane and out-of-plane connections between these miniature, microfabricated building blocks are shown to withstand macroscale forces, and they provide enough force to enable leak-free sealing of their smooth surfaces without O-rings up to 120 kPa. The present blocks are roughly an order of magnitude smaller in their linear dimensions than those of [11,12], are batch-microfabricated, connect to each other mechanically via monolithically integrated interconnection features, and also enable microfluidic connection. As the blocks provide effective mechanical and fluid connections via compact structures, it is possible to make modular microfluidic elements in which the functional microfluidic volume (e.g., a square microwell 0.5-mm across and 0.2-mm high) is a significant fraction of the total device volume including interconnections (1 × 1 × 0.9 mm). The present advances are enabled by the first demonstration of a hybrid molding/photolithographic process. As a first step towards miniature, microfabricated platforms for microfluidics, the compatibility of our modular microwell arrays (Figure 3-3b,c) and coupled microwells with cell culture, cell migration, and drug toxicity evaluation via a miniaturized in vitro platform was shown. In the future, the interlocks may be integrated into elements with various shapes, compositions, and embedded functions for applications in actuation, more complex microfluidics, and microrobotics.
